# Clinical Utility and Patient-Level Analysis of IDDSI Level 1 (Slightly Thick) in Oropharyngeal Dysphagia: A Strategy to Mitigate Over-Thickening and Support Hydration

**DOI:** 10.3390/nu18162703

**Published:** 2026-08-19

**Authors:** Jae Woo Kim, Seung Yoon Choi, Ji Woo Lee, Seong Ho Jang, Seung Hoon Han, Jae Hyeon Park, Yeo Joon Yun

**Affiliations:** 1 Department of Rehabilitation Medicine, Hanyang University Medical Center, Seoul 04763, Republic of Korea; nhlsakic@naver.com (J.W.K.); wazsy28@naver.com (S.Y.C.); bakusaiga@naver.com (J.W.L.); 2 Department of Rehabilitation Medicine, Hanyang University College of Medicine, Hanyang University Guri Hospital, Guri 11923, Republic of Korea; systole77@daum.net (S.H.J.); stephan72@hanmail.net (S.H.H.); 3 Department of Rehabilitation Medicine, Hanyang University College of Medicine, Seoul 04763, Republic of Korea

**Keywords:** dysphagia, IDDSI level 1, slightly thick liquids, thickened liquids, nutrition, hydration, videofluoroscopic swallowing study, penetration–aspiration scale, stroke, number needed to treat

## Abstract

**Background/Objectives:** The International Dysphagia Diet Standardisation Initiative (IDDSI) introduced Level 1 (slightly thick) as a new intermediate consistency between thin liquid (Level 0) and mildly thick (Level 2). Although over-thickening is associated with reduced hydration and impaired medication absorption, the specific clinical utility of Level 1 has not been quantified. We aimed to determine the clinical benefit of Level 1 and identify the proportion of dysphagia patients for whom Level 1 is the minimum sufficient consistency. **Methods:** We retrospectively analyzed 163 consecutive patients undergoing a videofluoroscopic swallowing study (VFSS) in a single rehabilitation department, including a pre-specified stroke subgroup (*n* = 87). A de-escalation protocol was used (IDDSI Level 3 → 2 → 1 → 0) with stepwise bolus volumes (2–3 mL followed by 5 mL at each consistency), and testing was discontinued at thinner consistencies once aspiration (Penetration–Aspiration Scale [PAS] ≥ 6) was observed. PAS values at untested thinner levels following aspiration were imputed (assigned an assumed value reflecting the expected clinical outcome) as 8 in the primary analysis. We quantified Level 1 effectiveness using absolute risk reduction (ARR), relative risk reduction (RRR), and number needed to treat (NNT). **Results:** In paired analyses, Level 1 reduced aspiration versus Level 0 by 12.0 percentage points (RRR 31.5%, NNT 8.4) in the full cohort and 13.7 percentage points (RRR 38.5%, NNT 7.3) in the stroke subgroup. Incremental escalation from Level 1 to Level 2 yielded only modest additional benefit (incremental NNT 18.4 and 38.5). Patient-level classification identified 13.7% (full) and 16.5% (stroke) of patients as “Level 1-sufficient”, defined as unsafe on thin liquid but safe on Level 1. **Conclusions:** IDDSI Level 1 provides substantial aspiration protection compared to thin liquid (NNT ≈ 7–8) with diminishing returns from further escalation, supporting Level 1 as a clinically useful and underutilized first-line thickened liquid option that may help avoid the nutritional and hydration drawbacks of over-thickening.

## 1. Introduction

Oropharyngeal dysphagia is a common and clinically important condition affecting an estimated 30–65% of hospitalized stroke patients and a substantial proportion of older adults in rehabilitation settings [1,2]. Aspiration is the most feared complication and a well-established risk factor for aspiration pneumonia, prolonged hospitalization, malnutrition, and mortality. For patients with unsafe swallowing on thin liquids, the cornerstone of management is dietary modification, specifically, increasing bolus consistency to slow oropharyngeal transit and reduce airway invasion [3,4,5]. However, indiscriminate increases in bolus consistency carry their own well-recognized drawbacks, creating a central clinical tension between airway safety and the nutritional costs of thickening, which motivated the present study.

The International Dysphagia Diet Standardisation Initiative (IDDSI), introduced in 2017, harmonized previously heterogeneous classification schemes into a single framework spanning eight levels (0–7) [6]. A critical refinement was the introduction of Level 1 (slightly thick), an intermediate consistency between thin liquid (Level 0) and mildly thick (Level 2). Level 1 did not exist as a discrete category in the previous National Dysphagia Diet (NDD), in which the lowest thickened consistency was nectar-thick (corresponding to IDDSI Level 2). The introduction of Level 1 opened the possibility of a more graded, minimal-intervention approach to thickened liquid prescription.

Optimal consistency selection in dysphagia management must balance airway safety against the nutritional and hydration consequences of over-thickening. Higher-consistency liquids reduce aspiration risk but are also associated with increased pharyngeal residue, reduced palatability, decreased oral fluid intake leading to dehydration, impaired bioavailability of solid-dose medications, and diminished quality of life [7,8,9]. These nutritional and pharmacological drawbacks are particularly relevant in older and chronically ill populations, in whom adequate hydration, nutrient intake, and medication efficacy are central to overall health outcomes. Despite these established concerns, many clinicians default to prescribing Level 2 or above as the first-line thickening option, partly because the IDDSI framework is still being integrated into practice and partly because of a cautious bias toward a higher consistency as inherently safer. This default approach may unnecessarily expose patients to the nutritional risks of over-thickening when a lower consistency (Level 1) would have sufficed.

Published studies examining swallowing outcomes across IDDSI levels have generally presented group-level aspiration rates without quantifying the clinical benefit of Level 1 in effect-size terms that clinicians can use for decision-making. Metrics such as absolute risk reduction, relative risk reduction, and number needed to treat (NNT) are standard in therapeutic research and directly translate to clinical decisions but have rarely been applied in dysphagia dietary prescription. In addition, patient-level classification, identifying for whom Level 1 is specifically the minimum sufficient consistency, has not been systematically reported. A further analytic challenge arises from the de-escalation protocol used during VFSSs, in which thinner consistencies are not tested once aspiration occurs at a thicker one [10]. This protocol, rooted in patient safety, means that thinner-level PAS values are unobserved specifically for those patients who would be most likely to aspirate at those levels. Ignoring this structure and analyzing only observed values systematically underestimates aspiration risk at the thinnest, and therefore most clinically important, consistency. We emphasize that de-escalation testing itself is an appropriate, safety-motivated protocol, and our own study likewise uses this design. The limitation we highlight is specific to prior analyses that have relied on observed (complete-case) values only, a methodological gap recently articulated by Borders and Brates [10].

We therefore conducted a retrospective VFSS cohort analysis with two aims: first, to quantify the clinical benefit of IDDSI Level 1 relative to Level 0 and to examine the incremental benefit of further escalation to Level 2 using absolute risk reduction, relative risk reduction, and NNT; second, to classify patients by the minimum IDDSI level required for safe swallowing and describe the proportion of patients for whom Level 1 is the minimum sufficient consistency. A pre-specified subgroup analysis focused on stroke patients as the largest etiologically homogeneous population, and as the population in whom IDDSI-based thickened liquid prescription is most frequently applied in clinical practice, providing a natural comparator for the prior thickener prescription literature in stroke rehabilitation.

## 2. Materials and Methods

### 2.1. Study Design and Participants

This was a single-center retrospective observational study conducted at the Department of Rehabilitation Medicine, Hanyang University Guri Hospital. We reviewed all consecutive patients who underwent a VFSS for clinical evaluation of oropharyngeal dysphagia during the study period. Inclusion criteria included (1) adult patients (≥18 years) referred for a VFSS by a rehabilitation physician (2) with availability of complete PAS and residue scoring recorded at each IDDSI consistency level. The primary analytic cohort comprised all eligible patients (all-cause oropharyngeal dysphagia). A pre-specified subgroup analysis was performed in patients with documented stroke (ischemic infarction or non-traumatic intracerebral/subarachnoid hemorrhage). Among stroke patients, the median interval from stroke onset to VFSS was 25 days (interquartile range 11–88 days), with onset data available in 84 of 87 patients; the cohort was therefore predominantly in the subacute phase (70.2% subacute [7 days to 6 months], 13.1% acute [<7 days], 4.8% early chronic [6–12 months], and 11.9% chronic [>12 months]), reflecting the standard clinical practice in which post-stroke dysphagia is typically evaluated by rehabilitation services after acute stabilization. The study was conducted in accordance with the Declaration of Helsinki and was approved by the Institutional Review Board with a waiver of informed consent given the retrospective design and use of de-identified data.

### 2.2. VFSS Protocol and Outcome Measures

VFSSs were performed in the upright seated position using a standardized de-escalation protocol in which testing proceeded from thicker to thinner consistencies (IDDSI Level 3 → 2 → 1 → 0). For each consistency, an initial 2–3 mL bolus (approximately half of a standard teaspoon) was administered. If aspiration (PAS ≥ 6) was not observed at this volume, a subsequent 5 mL bolus (a full standard teaspoon) was given. Aspiration occurring at either bolus volume was classified as aspiration at that consistency level, and bolus volumes were not analyzed separately. Following the institutional safety policy, testing was discontinued at thinner consistencies once aspiration (PAS ≥ 6) was observed at a given level, since thinner boluses would be expected to carry at least an equivalent aspiration risk and further testing would add radiation exposure and risk of additional airway invasion without yielding new clinical information. Level 4 (extremely thick), being a fundamentally different consistency category (pureed rather than a pourable liquid), was tested independently of the de-escalation sequence and was administered as a single trial before de-escalation testing was completed rather than as part of the ordered Level 3 → 2 → 1 → 0 sequence. Boluses were prepared according to IDDSI flow test standards (IDDSI Gravity Flow Test, performed by trained nursing/dietary staff at the time of bolus preparation using an IDDSI-compliant syringe; flow was not independently re-verified for every individual bolus administered) and administered using a standard teaspoon under fluoroscopic guidance. All VFSS examinations were reviewed and scored by certified rehabilitation physicians, who, in some cases, were also the study authors. Scoring was not blinded to consistency level, as the tested consistency was directly visible on fluoroscopy, and formal inter-rater reliability (e.g., blinded, independent re-scoring) was not obtained in this retrospective cohort.

The primary safety outcome was the Penetration–Aspiration Scale (PAS) score at each IDDSI level. Aspiration was defined as PAS ≥ 6 and penetration as PAS 3–5 [11]. Pharyngeal (piriform sinus/posterior pharyngeal wall, excluding the valleculae) and vallecular (valleculae) residues were assessed separately as secondary outcomes using an institution-standardized ordinal 0–3 scale (0 = none; 1 = coating/trace; 2 = residue occupying a discrete but limited pool, e.g., a partially filled vallecula or pyriform sinus; 3 = residue occupying the greater part of the space, with pooling extending beyond a single pocket). This is an in-house scale rather than a validated instrument (see Limitations). Baseline swallowing assessments, including the Gugging Swallowing Screen (GUSS), Functional Oral Intake Scale (FOIS), Clinical Dysphagia Scale (CDS), and Korean Montreal Cognitive Assessment (MoCA-K), were obtained as part of the standard workup [12,13,14].

### 2.3. Clinical Effectiveness Metrics

We quantified the clinical effectiveness of Level 1 using paired analyses that explicitly reflect within-patient transitions. In plain terms, ARR is the absolute reduction in the percentage of patients aspirating when moved to a thicker consistency; RRR expresses that same reduction relative to the baseline aspiration rate; and NNT is the number of patients who would need to be prescribed the thicker consistency for one additional aspiration event to be prevented, a metric widely used in therapeutic intervention research but, to our knowledge, rarely applied to thickened liquid prescription. For each IDDSI pair of interest (Level 0 vs. Level 1; Level 1 vs. Level 2), we computed (1) the absolute risk reduction (ARR) in aspiration rate; (2) the relative risk reduction (RRR = ARR/higher-level aspiration rate × 100); and (3) the number needed to treat (NNT = 1/ARR), which represents the number of patients who must be prescribed the higher consistency level to prevent one additional aspiration. We additionally computed a paired discordance table (patients aspirating at one level but not the other) to identify the number of patients directly rescued by each consistency transition.

### 2.4. Patient-Level Classification

To identify the optimal IDDSI level for each individual patient, we classified each patient according to the lowest IDDSI level at which they demonstrated safe swallowing (PAS < 6). Categories were Safe at Level 0 (no thickening needed); Level 1-sufficient (unsafe at Level 0 but safe at Level 1, without requiring further escalation); Requires Level 2 (unsafe at Level 1 but safe at Level 2); Requires Level 3; Requires Level 4 only; or Unsafe at all tested levels. Patients with no PAS data at any IDDSI level (e.g., those on long-term tube feeding in whom oral trials were judged unsafe and not performed) were considered unclassifiable and excluded from this analysis. The “Level 1-sufficient” category is of primary clinical interest because it identifies patients who would be over-thickened if Level 2 were used as the default.

### 2.5. Statistical Analysis

Continuous variables are presented as mean ± SD or median (IQR), categorical variables as n (%). Before primary analysis, a database quality control check was performed to identify entries inconsistent with the de-escalation protocol (i.e., observed safe swallowing at a thinner consistency despite recorded aspiration at a thicker one)—a pattern that should not occur under the protocol and therefore signals a data entry rather than a clinical event. Four such discordant entries were identified and resolved through chart and image re-review by the attending physician, which confirmed data entry errors at Level 0 (i.e., the Level 0 finding had been mis-recorded, not a genuine instance of a thinner consistency being safe after aspiration at a thicker one); the corrected values were used in all subsequent analyses. The PAS across IDDSI Levels 0–4 was evaluated using the Friedman test, with pairwise Wilcoxon signed-rank tests and Holm–Bonferroni correction for multiple comparisons. Effect sizes are reported as rank-biserial r. Binary aspiration outcomes across levels were analyzed using Cochran’s Q test with pairwise exact McNemar tests.

The remainder of this section addresses the unobserved values in three steps: (1) explanation of why they occur and why they are not missing at random; (2) the clinical rationale for the primary imputation approach; and (3) two sensitivity analyses performed for comparison. Because the de-escalation protocol terminated testing at consistencies thinner than the level at which aspiration first occurred, PAS values at thinner levels were unavailable for a substantial proportion of patients. In the full cohort, the proportion of patients without observed PAS values was 31.9% at Level 0, 27.0% at Level 1, 18.4% at Level 2, 12.3% at Level 3, and 1.8% at Level 4; corresponding proportions in the stroke subgroup were 31.0%, 25.3%, 18.4%, 9.2%, and 3.4%. These unobserved values were not missing in the conventional statistical sense but rather reflected a deliberate clinical decision grounded in physiology: because aspiration had already occurred at a thicker bolus, a thinner bolus would be expected to carry at least the same risk, and further testing would offer no additional diagnostic value while increasing patient risk.

Complete-case analysis of such data would condition on the absence of aspiration at all consistencies and systematically underestimate aspiration risk at thinner ones. To address this, three pre-specified analyses were compared. The primary analysis imputed the PAS score at untested thinner levels as 8, reflecting the clinical expectation that a thinner bolus in a patient already aspirating at a thicker one would also result in aspiration. Two sensitivity analyses were performed: a last-observation-carried-forward (LOCF) analysis, in which the PAS score at each untested thinner level was taken as the value observed at the thickest tested level (yielding a lower-bound estimate of aspiration at thinner levels); and a complete-case analysis using only observed values (the conventional approach, reported for comparison). Because residue (unlike aspiration) was not the basis for protocol discontinuation, and no safety threshold analogous to PAS ≥ 6 applies to residue scoring, pharyngeal and vallecular residue scores at untested consistencies were not imputed; complete-case data were used for residue outcomes.

Analyses were performed separately in the full cohort (primary) and the stroke subgroup (pre-specified). A two-sided *p* < 0.05 was considered significant. All analyses were performed in Python (version 3; Python Software Foundation, Wilmington, DE, USA) using scipy.stats. (SciPy community, https://scipy.org).

## 3. Results

### 3.1. Patient Characteristics

A total of 163 consecutive patients were included in the primary analytic cohort (mean age 72.5 ± 13.2 years; 85 male [52.1%]). The stroke subgroup comprised 87 patients (mean age 71.1 ± 12.1 years; 47 male [54.0%]), representing 53.4% of the full cohort. Non-stroke etiologies included aspiration pneumonia without documented stroke (n = 20), traumatic brain injury (n = 10), Parkinson’s disease (n = 8), neuromuscular disease (n = 4), malignancy (n = 8), and other conditions (n = 26). Baseline clinical swallowing assessments are summarized in Table 1.

### 3.2. Aspiration Rates Across IDDSI Levels

In the primary (imputation-based) analysis, aspiration rates decreased monotonically with increasing consistency. In the full cohort, aspiration rates were 38.2%, 25.2%, 19.2%, 16.9%, and 8.1% at Levels 0, 1, 2, 3, and 4, respectively (Friedman *p* < 0.001). The stroke subgroup showed the same pattern (36.5%, 20.8%, 17.5%, 16.9%, 6.0%; Friedman *p* < 0.001). Detailed pairwise comparisons are presented in Table 2 and Table S1, with the overall pattern shown in Figure S1. Aspiration rates decreased monotonically across IDDSI levels, from 38.2% (Level 0) to 8.1% (Level 4) in the full cohort (and 36.5% to 6.0% in the stroke subgroup).

### 3.3. Clinical Benefit of Level 1 Versus Level 0

The magnitude of the clinical benefit conferred by Level 1 over thin liquid is summarized in Table 3. In the full cohort, escalating from Level 0 to Level 1 produced an absolute aspiration risk reduction of 12.0 percentage points, corresponding to a 31.5% relative risk reduction and a number needed to treat (NNT) of 8.4; that is, one aspiration event is prevented for every 8–9 patients whose diet is upgraded from thin to slightly thick. In the stroke subgroup, the effect was even larger: ARR: 13.7 percentage points, RRR: 38.5%, NNT: 7.3. In paired analyses, 17 of 142 full-cohort patients (12.0%) and 10 of 73 stroke patients (13.7%) were aspirators at Level 0 but non-aspirators at Level 1, providing direct evidence of patient-level rescue by minimal thickening. Following the database correction described in the Methods section, no paradoxical pairs (Level 0 safe but Level 1 aspirating) remained.

### 3.4. Incremental Benefit of Level 2 over Level 1

In contrast, further escalation from Level 1 to Level 2 produced substantially smaller incremental benefits (Table 3). In the full cohort, the additional ARR was 5.4 percentage points with an incremental NNT of 18.4; that is, one additional patient was rescued from aspiration for every 18–19 patients upgraded from Level 1 to Level 2. In the stroke subgroup, this effect was further attenuated, with an ARR of 2.6 percentage points and an incremental NNT of 38.5, meaning approximately 39 stroke patients would need to be upgraded to Level 2 to prevent one additional aspiration event beyond what Level 1 already accomplishes. Paired discordance analysis identified 10 of 147 full-cohort patients (6.8%) and 4 of 77 stroke patients (5.2%) who were unsafe on Level 1 but safe on Level 2, representing the true incremental beneficiaries of the Level 1–Level 2 transition.

### 3.5. Patient-Level Classification Identifies the Level 1-Sufficient Group

**Figure 1:**
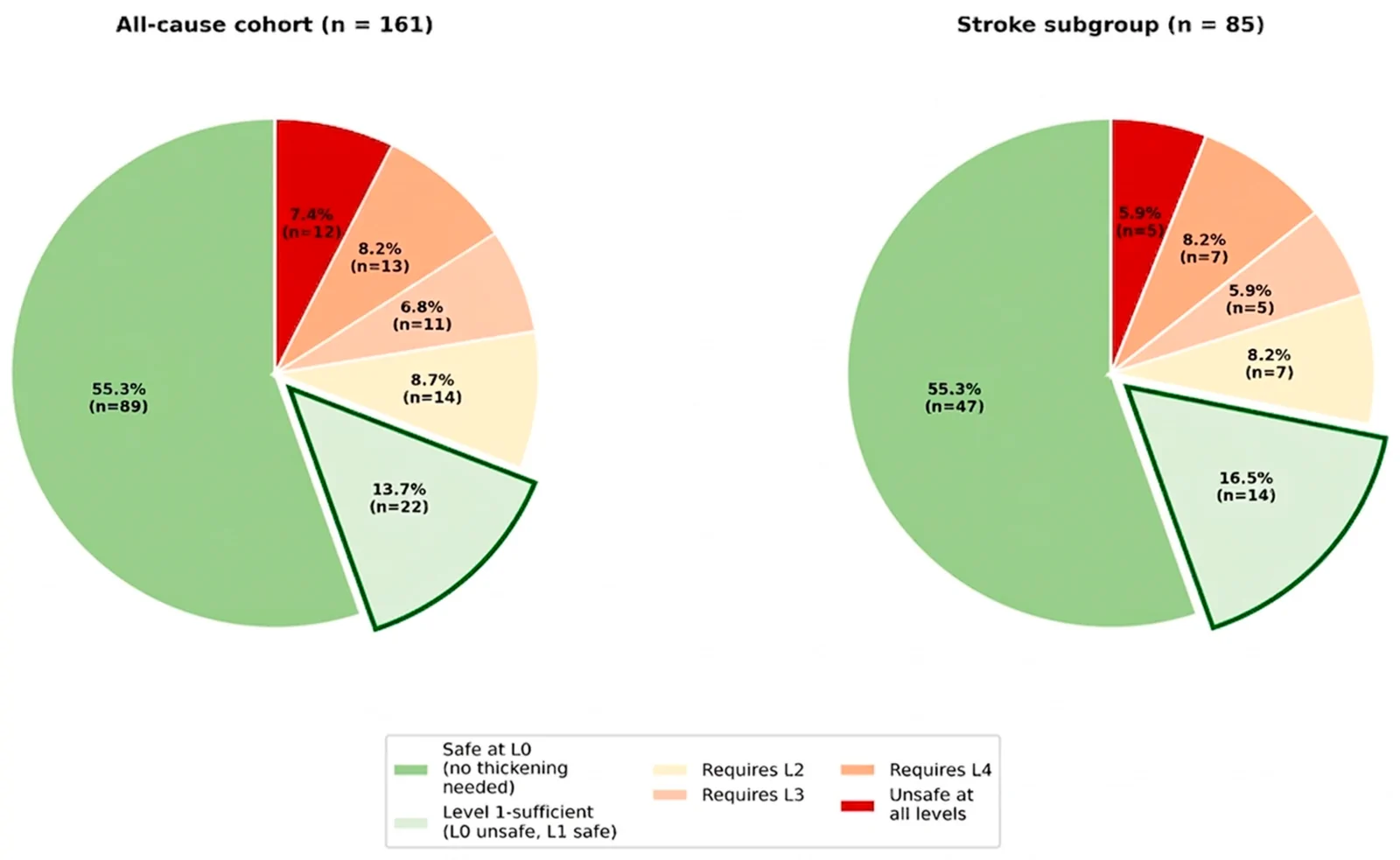
Patient-level classification by the minimum IDDSI level required for safe swallowing. Proportions of patients classified by the minimum IDDSI level at which they demonstrated safe swallowing. The Level 1-sufficient category (exploded slice, dark-green border) comprises patients unsafe on thin liquid but safe on Level 1 without requiring further escalation: 13.7% of the full cohort and 16.5% of the stroke subgroup.

Classification of each patient by the minimum IDDSI level required for safe swallowing revealed clinically distinct groups (Table 4, Figure 1). Of the 163 patients in the full cohort and 87 patients in the stroke subgroup, 161 and 85, respectively, had at least one IDDSI level with available PAS data and were therefore classifiable; the remaining two patients (both stroke patients on long-term tube feeding) were not orally assessed and were excluded from this analysis. The largest group comprised patients already safe on thin liquid (Level 0; 55.3% of the full cohort, 55.3% of stroke patients). Among patients who required thickening, the largest subset was the Level 1-sufficient group: 22 of 161 (13.7%) in the full cohort and 14 of 85 (16.5%) in the stroke subgroup. These patients were unsafe on Level 0 but safe on Level 1 and did not require further consistency escalation. Smaller proportions required Level 2 (8.7%/8.2%), Level 3 (6.8%/5.9%), or Level 4 only (8.1%/8.2%), and 7.5%/5.9% were unsafe even at Level 4. This patient-level view makes it clear that a non-trivial fraction of patients, approximately one in six stroke patients, would be unnecessarily over-thickened if Level 2 or above were used as the default starting consistency, exposing them to avoidable hydration and medication absorption concerns.

### 3.6. Pharyngeal and Vallecular Residues

**Figure 2:**
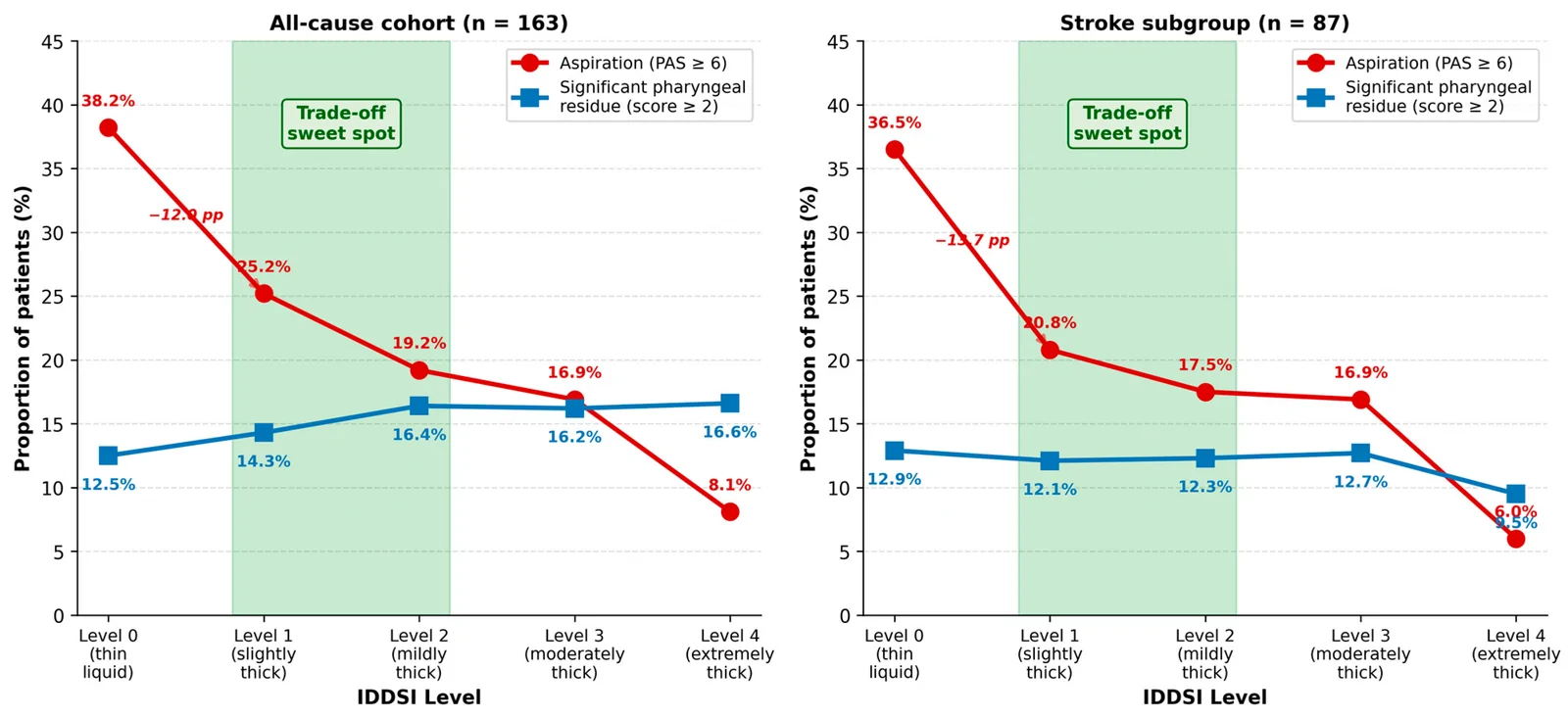
Safety–efficiency trade-off across IDDSI levels. Consistency increases while residue increases modestly, producing a trade-off. The shaded zone indicates the Level 0 → Level 1 → Level 2 transition region where the Level 0 → Level 1 step provides the most favorable balance.

Residue outcomes were analyzed as secondary endpoints using complete-case data (Table 5) to examine whether post-swallow residue increases as prescribed consistency increases (a dose–response question analogous to the aspiration analysis above). In the full cohort, residues in the piriform sinuses/pharyngeal walls showed a small but statistically significant increase across IDDSI levels (Friedman *p* = 0.002), with the proportion of patients with clinically significant residue (score ≥ 2) rising from 12.5% at Level 0 to 16.6% at Level 4. Vallecular residue showed no significant pattern across levels (*p* = 0.43). In the stroke subgroup, pharyngeal residue similarly reached statistical significance (*p* = 0.006) but with minimal between-level variation (proportion with significant residue ranging 9.5–12.9%), and vallecular residue did not differ significantly (*p* = 0.14). Viewed against the aspiration findings, these modest residue increases (particularly the 2.1-percentage-point rise in significant pharyngeal residue between Level 1 and Level 2 in the full cohort against the 6.0-percentage-point reduction in aspiration) illustrate the safety–efficiency trade-off inherent to consistency escalation (Figure 2). Notably, residue at Level 1 was indistinguishable from that at Level 0 (pharyngeal 14.3% vs. 12.5% in full cohort; 12.1% vs. 12.9% in stroke), indicating that the aspiration benefit of Level 1 is not offset by a meaningful residue penalty. The overall magnitude of residue differences was small and should be interpreted in light of the ordinal scale used (see Limitations).

### 3.7. Sensitivity Analyses

The primary conclusions were robust under alternative analytic assumptions. In the LOCF sensitivity analysis, aspiration rates were marginally attenuated but the monotonic pattern and the large Level 0 → Level 1 benefit were preserved. In the complete-case sensitivity analysis, aspiration rates at thinner levels were substantially underestimated (e.g., Level 0 aspiration was 18.9% in stroke patients in complete-case analysis versus 36.5% in the primary imputation-based analysis), confirming that complete-case analysis is inadequate for de-escalation protocols and would materially mask the clinical benefit of Level 1.

## 4. Discussion

This study provides the first systematic quantification of the clinical effectiveness of IDDSI Level 1 in an unselected oropharyngeal dysphagia cohort. Three findings have direct implications for clinical practice: First, Level 1 confers substantial aspiration-protective benefit over thin liquid, with a number needed to treat of approximately 8 in the full cohort and 7 in stroke patients, which is comparable to or better than many accepted medical interventions. Second, further escalation from Level 1 to Level 2 yields diminishing returns, with an incremental NNT of 18 in the full cohort and 39 in stroke patients. Third, the Level 1-sufficient patient population, those for whom Level 1 is the optimal consistency, constitutes approximately one in seven all-cause dysphagia patients and one in six stroke patients in our cohort. Collectively, these findings argue that Level 1 should be considered as a viable and underutilized first-line thickened liquid option rather than being bypassed in favor of higher consistencies.

The magnitude of the Level 1 benefit is clinically important. The 31.5–38.5% relative risk reduction in aspiration, accompanied by the paired rescue of 12–14% of all studied patients, establishes Level 1 as a meaningful therapeutic intervention rather than a marginal consistency change. That 17 full-cohort patients (and 10 stroke patients) were directly converted from aspirators at Level 0 to non-aspirators at Level 1 provides person-level evidence that minimum thickening is a sufficient intervention for a specific clinical subgroup. Conversely, the incremental NNT of 38.5 in stroke patients for escalation to Level 2 quantifies a common clinical overreach: routine escalation to Level 2 would provide benefit to one additional patient for every 39 unnecessarily thickened. This trade-off framing should not be read as implying that Level 2 and above are rarely needed; approximately 30% of classifiable patients in our cohort required Level 2 or higher consistency, or remained unsafe despite thickening, and continue to rely on escalation for airway safety. Our findings support Level 1 as an important first-line option to be confirmed by instrumental assessment, not as a substitute for higher consistencies in patients with more severe dysphagia, who may derive greater benefit from Levels 2–4.

These findings have particular nutritional and pharmacological significance. Higher-consistency liquids are well documented as reducing voluntary fluid intake, contributing to dehydration, which is already a major concern in older adults with dysphagia. Cichero and colleagues demonstrated that thickened liquids retard medication release at viscosities as low as 150 mPa·s, with the effect amplified at higher viscosities; provision of mildly thick and thicker fluids is therefore particularly likely to compromise oral medication efficacy [7]. Reduced palatability, increased pharyngeal residue, and diminished quality of life are additional consequences of over-thickening that disproportionately affect patients prescribed higher IDDSI levels [15]. By identifying the substantial subset of patients (16.5% of stroke patients) for whom Level 1 is the minimum sufficient consistency, our findings suggest a concrete opportunity to mitigate these nutritional and pharmacological risks without compromising airway safety. Avoiding unnecessary escalation to Level 2 or above in this subgroup may improve hydration status, medication efficacy, and dietary acceptance. We emphasize that these potential benefits were not directly measured in the present study and are extrapolated from the prior literature rather than demonstrated in this cohort; prospective studies incorporating hydration, medication level, and quality-of-life outcomes are needed to confirm that avoiding unnecessary escalation translates into a measurable clinical benefit (see Limitations).

The patient-level classification refines this picture further. Prior to the IDDSI framework, Level 1-sufficient patients would have been prescribed the nectar-thick consistency of the NDD (equivalent to IDDSI Level 2) despite not requiring that degree of thickening. The introduction of Level 1 thus fills a genuine clinical gap, and our data quantify for whom and to what extent. Equally important is the fact that the majority of patients requiring thickening (those who need Level 2 or above) are adequately served by higher consistencies, so Level 1 is not a universal recommendation but rather a first-line option whose adequacy should be confirmed by instrumental assessment.

Our findings extend a small but growing body of literature on IDDSI-stratified swallowing outcomes. Steele and colleagues recently provided reference videofluoroscopic data across IDDSI Levels 0–4 in healthy adults and individuals with suspected dysphagia, demonstrating that aspiration risk decreases with increasing consistency in a manner broadly consistent with our results [16,17]. However, those studies used ascending-order testing protocols and were primarily aimed at generating reference values rather than quantifying clinical effectiveness. The earlier Korean rehabilitation literature, including studies on thickener prescription and compliance after stroke, has documented the importance of consistency modification but has not stratified outcomes across the discrete IDDSI levels [18]. To our knowledge, the application of effect-size measures such as NNT and ARR to IDDSI Level 1, together with patient-level classification, has not been previously reported in a clinical dysphagia cohort. A systematic review by Borders and Brates highlighted a field-wide lack of methodological rigor in handling unobserved PAS values across multiple bolus consistencies, particularly under de-escalation protocols; our analytic approach directly addresses this concern [10]. The clinical importance of Level 1 has been emphasized in recent expert commentary describing its potential to reduce over-thickening, but our work is among the first to quantitatively demonstrate its utility using metrics relevant to clinical decision-making.

A methodological strength of this study is the explicit treatment of the structure of unobserved values inherent in VFSS de-escalation testing. Because thinner consistencies are not tested after aspiration occurs at a thicker one (a safety-driven clinical decision rather than a random data loss), complete-case analysis systematically underestimates aspiration risk at thinner levels, which are precisely the levels at which safety assessment matters most. The magnitude of this bias in our data is substantial: in the stroke subgroup, complete-case analysis estimated Level 0 aspiration at 18.9%, whereas principled imputation reflecting the clinical reality of de-escalation yielded 36.5%. This bias would obscure the very Level 1 benefit that our analysis identifies. We recommend that future IDDSI-stratified VFSSs routinely report imputation-based sensitivity analyses alongside complete-case estimates.

### Study Limitations

This study has several limitations, grouped by theme. With respect to design and generalizability: It is a single-center retrospective analysis, which limits generalizability; although our cohort represents tertiary-center dysphagia referrals, practice patterns and case mix may differ elsewhere. The primary cohort was also etiologically heterogeneous. We partially addressed this with the pre-specified stroke subgroup, which showed even more favorable Level 1 benefit estimates. Among stroke patients, the cohort was predominantly subacute (median onset-to-VFSS interval 25 days, with 70% in the subacute phase), reflecting standard rehabilitation practice in which patients are referred after acute stabilization; our findings may therefore be most directly applicable to subacute-phase stroke patients, and phase-stratified analyses to compare effectiveness across acute, subacute, and chronic phases represent an important direction for future research. In addition, the Level 1-sufficient proportion we report (13.7%/16.5%) reflects our cohort’s specific severity distribution and should not be assumed to generalize directly to populations with a different severity profile; additional analyses stratified by baseline severity (e.g., GUSS, FOIS, CDS, or baseline PAS) were not performed within the scope of the current revision and represent an important direction for future work.

With respect to measurement and reliability: Inter-rater reliability for PAS and residue scoring was not formally quantified in this cohort, although all scoring was performed by certified rehabilitation physicians using standardized criteria (Section 2.2). Prospective work should incorporate formal inter-rater reliability assessments. Although a pre-analysis quality control review identified and corrected four data entry errors at Level 0, a small number of paradoxical pairs at the Level 1–Level 2 transition (1.4–2.6% of paired comparisons) remained. These likely reflect the within-session measurement variability of the PAS around the penetration–aspiration cut-off and consistency-specific sensorimotor patterns occasionally observed in brainstem stroke and were retained in the analysis to preserve fidelity to observed data. The residue scale used at our institution is a four-point ordinal scale (0–3), an in-house scale rather than a validated instrument, which is likely subject to a ceiling effect and limited sensitivity; more granular scales such as the Yale Pharyngeal Residue Severity Rating Scale may capture residue trends more sensitively.

With respect to the scope of outcomes measured: We used VFSS surrogates for safety. Mapping to prospective clinical outcomes such as aspiration pneumonia, hydration status, medication levels, and long-term quality of life requires prospective follow-up, which was beyond the scope of this analysis, and the hydration, medication bioavailability, palatability, and quality-of-life benefits we attribute to avoiding over-thickening in the Discussion are drawn from the prior literature rather than directly measured in this cohort. The imputation strategy is a worst-case assumption chosen to reflect the clinical meaning of the de-escalation protocol; sensitivity analyses confirmed robustness but alternative imputations may yield somewhat different magnitude estimates. NNT estimates are sensitive to underlying aspiration prevalence, which, in our cohort, reflects a population referred for VFSSs; NNTs in lower-risk populations would be higher. Finally, we did not formally characterize silent aspiration patterns; however, given that silent aspiration is known to be common in neurogenic dysphagia, clinicians prescribing any thickened liquid level should remain vigilant and maintain periodic instrumental or clinical reassessment.

Notwithstanding these limitations, our findings support a practical clinical framework for IDDSI-based thickened liquid prescription that balances airway safety with nutritional outcomes. For patients with unsafe swallowing on thin liquid, Level 1 should be considered first, with confirmation of safety by instrumental assessment. Patients demonstrated to be safe on Level 1 by a VFSS should be maintained on Level 1 rather than prophylactically escalated, thereby preserving palatability, hydration, and medication efficacy. Patients found to be unsafe on Level 1 should be escalated stepwise to higher consistencies as needed. Importantly, our findings also indicate that a substantial proportion of patients (approximately 30%) required IDDSI Level 2 or higher to achieve safe swallowing, underscoring that Level 1 is not sufficient for all individuals. Therefore, thickened liquid prescription should remain individualized based on instrumental swallowing assessment rather than a uniform preference for lower consistency. Prospective multi-center studies incorporating long-term nutritional and clinical outcomes are needed to confirm and refine this framework.

## 5. Conclusions

In a retrospective IDDSI-stratified VFSS cohort of 163 oropharyngeal dysphagia patients, including 87 stroke patients, IDDSI Level 1 provided clinically meaningful aspiration protection over thin liquid (NNT 7–8), with substantially smaller incremental benefit from further escalation to Level 2 (incremental NNT 18–39). Approximately one in seven all-cause dysphagia patients and one in six stroke patients were Level 1-sufficient, defined as unsafe on thin liquid but adequately protected by Level 1. Adoption of Level 1 as a first-line option in appropriate patients might help avoid the nutritional and pharmacological drawbacks of over-thickening, including reduced hydration and impaired medication absorption, while preserving airway safety. These findings support incorporating Level 1 as a clinically useful first-line option within an individualized IDDSI-based thickened liquid prescription strategy while recognizing that many patients require higher consistency levels to achieve safe swallowing.

## Figures and Tables

**Table 1:** Baseline characteristics of the study cohort.

Variable	Full Cohort (n = 163)	Stroke Subgroup (n = 87)
Age, years (mean ± SD)	72.5 ± 13.2	71.1 ± 12.1
Sex: Male, n (%)	85 (52.1)	47 (53.4)
MoCA-K, median (IQR)	9.0 (1.0–17.0) [n = 135]	9.0 (2.0–17.5) [n = 75]
Indirect GUSS, median (IQR)	4.0 (2.2–5.0) [n = 70]	4.0 (2.0–5.0) [n = 45]
Direct GUSS, median (IQR)	0.0 (0.0–3.8) [n = 70]	0.0 (0.0–4.0) [n = 45]
FOIS, median (IQR)	5.0 (1.0–6.0) [n = 70]	4.0 (1.0–5.0) [n = 45]
CDS, median (IQR)	15.0 (8.5–32.0) [n = 163]	15.0 (6.5–30.0) [n = 87]
Onset-to-VFSS interval, days (median, IQR)	—	25 (11–88) [n = 84]
Stroke phase at VFSS, n (%) *	—	
Acute (<7 days)	—	11 (13.1%)
Subacute (7 days–6 months)	—	59 (70.2%)
Early chronic (6–12 months)	—	4 (4.8%)
Chronic (>12 months)	—	10 (11.9%)

GUSS: Gugging Swallowing Screen; FOIS: Functional Oral Intake Scale; CDS: Clinical Dysphagia Scale; MoCA-K: Korean Montreal Cognitive Assessment; IQR: interquartile range; SD: standard deviation. Values in brackets indicate the number of patients with available data. * Phase percentages calculated among 84 stroke patients with documented onset date; onset was unknown in 3 patients.

**Table 2:** Aspiration rates (PAS ≥ 6) by IDDSI level: primary (imputation-based) analysis.

	Full Cohort (n = 163)			Stroke Subgroup (n = 87)		
IDDSI Level	n	Asp n	Asp %	n	Asp n	Asp %
Level 0	144	55	38.2%	74	27	36.5%
Level 1	147	37	25.2%	77	16	20.8%
Level 2	151	29	19.2%	80	14	17.5%
Level 3	154	26	16.9%	83	14	16.9%
Level 4	160	13	8.1%	84	5	6.0%

PAS: Penetration–Aspiration Scale. Friedman tests across Levels 0–4: *p* < 0.001 in both cohorts.

**Table 3:** Clinical benefit of IDDSI Level 1: number needed to treat and paired discordance analysis.

Metric	Definition	Full Cohort	Stroke Subgroup
Level 0 vs. Level 1: benefit of minimal thickening			
Paired n	Patients with both L0 and L1 observed/imputed	142	73
ARR	L0−L1 aspiration rate	12.0 pp	13.7 pp
RRR	ARR/L0 rate × 100	31.5%	38.5%
NNT	Patients upgraded to L1 to prevent 1 aspiration	8.4	7.3
L1 rescue count	Patients aspirating at L0 but not at L1	17 (12.0%)	10 (13.7%)
Paradoxical count	Patients safe at L0 but aspirating at L1	0 (0%)	0 (0%)
Level 1 vs. Level 2: incremental benefit of further thickening			
Paired n	Patients with both L1 and L2 observed/imputed	147	77
Additional ARR	L1−L2 aspiration rate	5.4 pp	2.6 pp
Incremental NNT	Patients upgraded to L2 to prevent 1 additional aspiration	18.4	38.5
L2 additional rescue	Patients aspirating at L1 but not at L2	10 (6.8%)	4 (5.2%)
Paradoxical count	Patients safe at L1 but aspirating at L2	2 (1.4%)	2 (2.6%)

ARR: absolute risk reduction, expressed in percentage points (pp); RRR: relative risk reduction (expressed as %); NNT: number needed to treat. Note that percentage points (pp) represent the absolute difference between two percentages, whereas % denotes a relative proportion. Paired analyses use the primary (imputation-based) analysis.

**Table 4:** Patient-level classification by the minimum IDDSI level required for safe swallowing.

Category	Definition	Full Cohort n (%)	Stroke n (%)
Safe at Level 0	No thickening required; safe on thin liquid	89 (55.3%)	47 (55.3%)
Level 1-sufficient *	Unsafe at L0, safe at L1: Level 1 is the minimum required consistency	22 (13.7%)	14 (16.5%)
Requires Level 2	L1 still unsafe; L2 provides safe swallow	14 (8.7%)	7 (8.2%)
Requires Level 3	L2 unsafe; L3 provides safe swallow	11 (6.8%)	5 (5.9%)
Requires Level 4 only	Unsafe until extremely thick (pureed) consistency	13 (8.1%)	7 (8.2%)
Unsafe at all levels tested	Aspirated at all levels including L4	12 (7.5%)	5 (5.9%)
Total classified		161	85

* The Level 1-sufficient category represents patients who specifically benefit from the availability of Level 1 as a distinct consistency. Without Level 1, these patients would be escalated to Level 2 or higher unnecessarily. Totals are less than the parent cohorts because two patients (both with documented stroke and on long-term tube feeding at the time of the VFSS) had no oral PAS data at any IDDSI level and were therefore unclassifiable. Based on the primary (imputation-based) analysis.

**Table 5:** Pharyngeal and vallecular residue scores across IDDSI levels: complete-case analysis.

Residue Location	IDDSI	n	Mean ± SD	Median (IQR)	Score ≥ 2, n (%)
Full cohort (n = 163)					
Pharyngeal	Level 0	112	0.92 ± 0.57	1.0 (1.0–1.0)	14 (12.5%)
	Level 1	119	0.92 ± 0.60	1.0 (1.0–1.0)	17 (14.3%)
	Level 2	134	0.96 ± 0.61	1.0 (1.0–1.0)	22 (16.4%)
	Level 3	142	0.94 ± 0.64	1.0 (1.0–1.0)	23 (16.2%)
	Level 4	157	0.96 ± 0.65	1.0 (1.0–1.0)	26 (16.6%)
Vallecular	Level 0	112	1.17 ± 0.52	1.0 (1.0–1.0)	26 (23.2%)
	Level 1	120	1.19 ± 0.51	1.0 (1.0–1.0)	29 (24.2%)
	Level 2	134	1.22 ± 0.54	1.0 (1.0–2.0)	36 (26.9%)
	Level 3	142	1.23 ± 0.56	1.0 (1.0–2.0)	40 (28.2%)
	Level 4	157	1.23 ± 0.61	1.0 (1.0–2.0)	43 (27.4%)
Stroke subgroup (n = 87)					
Pharyngeal	Level 0	62	0.87 ± 0.61	1.0 (0.2–1.0)	8 (12.9%)
	Level 1	66	0.85 ± 0.61	1.0 (0.0–1.0)	8 (12.1%)
	Level 2	73	0.85 ± 0.62	1.0 (0.0–1.0)	9 (12.3%)
	Level 3	79	0.84 ± 0.67	1.0 (0.0–1.0)	10 (12.7%)
	Level 4	84	0.80 ± 0.64	1.0 (0.0–1.0)	8 (9.5%)
Vallecular	Level 0	62	1.13 ± 0.50	1.0 (1.0–1.0)	12 (19.4%)
	Level 1	66	1.12 ± 0.48	1.0 (1.0–1.0)	12 (18.2%)
	Level 2	73	1.11 ± 0.49	1.0 (1.0–1.0)	13 (17.8%)
	Level 3	79	1.11 ± 0.53	1.0 (1.0–1.0)	16 (20.3%)
	Level 4	84	1.05 ± 0.49	1.0 (1.0–1.0)	12 (14.3%)

Friedman tests across Levels 0–4: Pharyngeal residue (full cohort *p* = 0.002, stroke subgroup *p* = 0.006). Vallecular residue (full cohort *p* = 0.43, stroke subgroup *p* = 0.14). Residue was not imputed; complete-case data are shown. Scale: 0 (none), 1 (coating/trace), 2 (residue occupying a discrete but limited pool), 3 (residue occupying the greater part of the space, with pooling beyond a single pocket).

## Data Availability

The data presented in this study are available on reasonable request from the corresponding author. The data are not publicly available due to patient privacy considerations.

## Supplementary Materials

The following supporting information can be downloaded at https://www.mdpi.com/article/10.3390/nu18162703/s1, Figure S1: Aspiration rate (PAS ≥ 6) across IDDSI levels: primary (imputation-based) analysis; Table S1: Pairwise comparisons of PAS scores between adjacent IDDSI levels: primary analysis.
