# Supplementary material for: Clinical Utility and Patient-Level Analysis of IDDSI Level 1 (Slightly Thick) in Oropharyngeal Dysphagia: A Strategy to Mitigate Over-Thickening and Support Hydration
Source: Nutrients. 2026 Aug 19;18(16):2703. doi: 10.3390/nu18162703 (PMC13516035; doi:10.3390/nu18162703)
Supplement: Supplementary file 1 [file nutrients-18-02703-s001.zip › nutrients-4385856-supplementary.pdf]

Supplementary Materials

Clinical Utility and Patient-Level Analysis of IDDSI Level 1 (Slightly Thick) in Oropharyngeal Dysphagia: A Strategy to Mitigate Over-Thickening and Support Hydration

Jae Woo Kim, Seung Yoon Choi, Ji Woo Lee, Seong Ho Jang, Seung Hoon Han, Jae Hyeon Park * and Yeo Joon Yun *

Table S1

Table S1. Pairwise comparisons of PAS scores between adjacent IDDSI levels: primary analysis.

Comparison	n paired	p (raw)	p (Holm-adj.)	Effect size r
**Full cohort (n = 163)**				
**L0 vs L1**	142	<0.001	**<0.001**	0.69
**L1 vs L2**	147	0.002	**0.004**	0.45
**L2 vs L3**	148	0.075	**0.075**	0.33
**L3 vs L4**	154	<0.001	**<0.001**	0.69
**Stroke subgroup (n = 87)**				
**L0 vs L1**	73	<0.001	**<0.001**	0.66
**L1 vs L2**	77	0.171	**0.343**	0.31
**L2 vs L3**	79	0.455	**0.455**	0.27
**L3 vs L4**	83	<0.001	**<0.001**	0.65

Wilcoxon signed-rank test with Holm-Bonferroni correction. Effect size r = rank-biserial correlation.

Figure S1

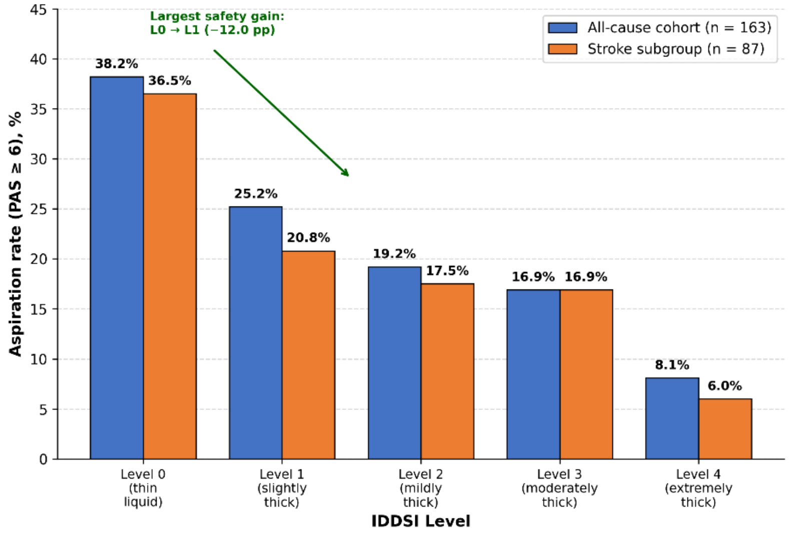

Figure 1. Aspiration rate (PAS ≥ 6) across IDDSI levels: primary (imputation-based) analysis. *Bars represent the percentage of patients with aspiration at each IDDSI level. Blue: all-cause cohort (n = 163); orange: stroke subgroup (n = 87). The monotonic dose-response pattern, with the largest reduction between Levels 0 and 1, is consistent across both cohorts.*
